# Characterization of the Molecular Determinants of Primary HIV-1 Vpr Proteins: Impact of the Q65R and R77Q Substitutions on Vpr Functions

**DOI:** 10.1371/journal.pone.0007514

**Published:** 2009-10-19

**Authors:** Guillaume Jacquot, Erwann Le Rouzic, Priscilla Maidou-Peindara, Marion Maizy, Jean-Jacques Lefrère, Vincent Daneluzzi, Carlos M. R. Monteiro-Filho, Duanping Hong, Vicente Planelles, Laurence Morand-Joubert, Serge Benichou

**Affiliations:** 1 Institut Cochin, Université Paris-Descartes, CNRS, UMR 8104, Paris, France; 2 Inserm, U567, Paris, France; 3 Institut National de la Transfusion Sanguine, Paris, France; 4 University of Utah, School of Medicine, Salt Lake City, Utah, United States of America; 5 Service de Bactériologie-Virologie, Centre hospitalo-universitaire de Saint-Antoine, APHP, Université Paris VI., Paris, France; Yale University, United States of America

## Abstract

Although HIV-1 Vpr displays several functions *in vitro*, limited information exists concerning their relevance during infection. Here, we characterized Vpr variants isolated from a rapid and a long-term non-progressor (LTNP). Interestingly, *vpr* alleles isolated from longitudinal samples of the LTNP revealed a dominant sequence that subsequently led to diversity similar to that observed in the progressor patient. Most of primary Vpr proteins accumulated at the nuclear envelope and interacted with host-cell partners of Vpr. They displayed cytostatic and proapoptotic activities, although a LTNP allele, harboring the Q65R substitution, failed to bind the DCAF1 subunit of the Cul4a/DDB1 E3 ligase and was inactive. This Q65R substitution correlated with impairment of Vpr docking at the nuclear envelope, raising the possibility of a functional link between this property and the Vpr cytostatic activity. In contradiction with published results, the R77Q substitution, found in LTNP alleles, did not influence Vpr proapoptotic activity.

## Introduction

Like for other 〈〈 auxiliary 〉〉 proteins of HIV-1, it has been suggested that Vpr is important *in vivo* for virus replication and pathogenesis (see Ref. [1] for review). The HIV-1 *vpr* gene encodes a 14 kDa protein and is one of the most highly conserved genes among primary isolates of human and simian immunodeficiency viruses (SIV). Vpr is found in HIV-1 virions, in infected cells, but also in sera and cerebro-spinal fluid of AIDS patients, suggesting that it participates in various aspects of the biology of HIV-1. The role of Vpr *in vivo* has been investigated in rhesus macaques infected with SIVmac, and it was initially shown that monkeys infected with SIV lacking the *vpr* and the related *vpx* genes displayed a lower virus burden and did not consistently develop immunodeficiency disease [2], [3]. Despite these observations, the exact contributions of Vpr during the natural course of infection are still elusive.

Several functions have been attributed to HIV-1 Vpr *in vitro*, including an effect on the reverse-transcription process, nuclear import of the viral DNA, cell cycle arrest at the G2/M transition, induction of apoptosis and transactivation of the HIV-1 LTR (see Ref. [1] for review). While its role in the reverse transcription process implies interaction with the nuclear form of the DNA repair enzyme UNG2 for modulation of the virus mutation rate [4]–[6], it was suggested that Vpr participates in the DNA nuclear import process through docking to the nuclear envelope (NE) by interaction with components of the nuclear pore complex [7]–[10], such as the nucleoporin hCG1. While these properties of Vpr have been shown to contribute to virus replication in non-dividing cells [10], [11], Vpr-induced G2-arrest, which is consequent to its interaction with the DCAF1 subunit of the Cul4a/DDB1 E3 ubiquitin ligase [12]–[17], may provide a favorable cellular environment for HIV-1 transcription [18]. In addition, the proapoptotic activity of Vpr has been postulated to contribute to the depletion of CD4+ T cells observed in infected patients.

Although numerous studies have demonstrated that mutations in HIV-1 Vpr could dramatically affect its known functions [19]–[21], these mutations were artificially created and did not represent the profile of naturally occurring mutations throughout the course of infection. While only a few genotypic analyses of the *vpr* alleles isolated from HIV-1 infected patients have been performed, there is no report regarding the functional characterization of primary Vpr variants during the course of infection. Nevertheless, an initial study has reported that a single substitution at the position Arg77 of HIV-1 Vpr (Arg77Gln) was found in a higher frequency in *vpr* alleles from long-term non-progressors (LTNPs) than in patients with AIDS [22]. While Mologni et al. confirmed this observation [23], several other groups did not find any association of the R77Q substitution with the prognosis of disease progression [24]–[28].

The present study aimed at analyzing the molecular and functional properties of primary Vpr proteins isolated from two infected patients, in terms of binding to its known cellular partners, docking at the NE, cytostatic and proapoptotic activities. In particular, a longitudinal analysis of Vpr variants isolated from a long-term non-progressor (LTNP) was performed.

## Results and Discussion

### Molecular analysis of *vpr* alleles

**Figure 1:**
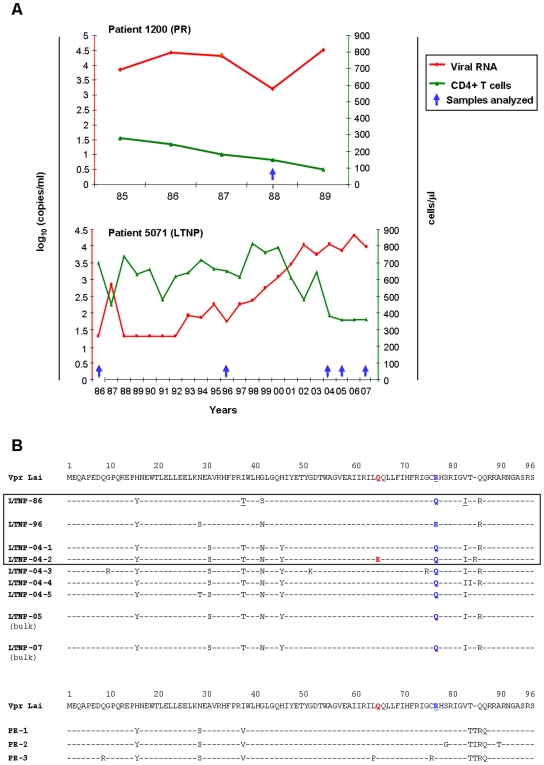
Description of patients and *vpr* alleles analyzed in the study. A) Immunological and viral profiles of the patients analyzed. These patients were selected from the HIV-1-infected patient cohort of the St-Antoine Hospital (Paris), and the samples were collected with written informed consent [45], [46]. The graphs show the time-course evolution of the blood CD4+ T cell counts (green curves) and plasmatic virus load (viral RNA, red curves); the PBMC samples analyzed in the present study are indicated by the blue arrows. B) Alignment of the amino acid sequences derived from DNA sequencing of the *vpr* alleles cloned from PBMC DNA samples. Excepted for LTNP-05 and LTNP-07 sequences that were obtained by direct sequencing of the bulk PCR fragments amplified from these samples, at least 40 independent clones were sequenced from each other samples. The sequences of the primary Vpr proteins are aligned with respect of the prototypic HIV-1*Lai* sequence (upper sequence). The *vpr* alleles from the LNTP (patient 5071) that were selected for subsequent functional analysis are in the box; the R77Q substitution identified in some *vpr* alleles is indicated in blue, while the Q65R substitution identified in the Vpr LTNP-04-2 is indicated in red.

Peripheral blood mononuclear cells (PBMCs) from the LTNP patient were sampled in 1986, 1996 and 2004 (*i.e.* 1, 11 and 19 years after infection) for longitudinal analysis of *vpr* alleles, and a single PBMC sample from a progressor patient was analyzed (Fig. 1A). V*pr* genes were amplified by PCR from the cellular DNA and cloned into a shuttle plasmid for genotypic analysis. DNA sequences from 40 independent clones recovered from two independent PCR were determined for each sample, allowing identification of the most represented *vpr* alleles from viral quasispecies. The relative frequency of the primary *vpr* alleles identified is recapitulated in Table 1. The first two samples analyzed from the LTNP, namely LTNP-86 and LTNP-96 (GenBank Accession numbers GU014240 and GU014241, respectively), showed a single *vpr* sequence sharing 95% a.a. identity (Fig. 1B), that subsequently evolved in a genetic diversity (LTNP-04-1 to -04-5; GenBank Accession numbers GU014242, GU014243, GU014244, GU014245 and GU014246, respectively) similar to that observed in the progressor patient (PR-1, PR-2 and PR-3; GenBank Accession numbers GU014237, GU014238 and GU014239, respectively). Notably, the sequence diversity of the LTNP-04 alleles correlated with an increase of the plasma viral load that paralleled a slight decline of the CD4+ T-cell count (Fig. 1A). This decrease of the CD4+ T-cells was still apparent in recent blood samples from 2005 and 2007, and bulk genotypic analysis of *vpr* from these samples (LTNP-05 and -07) showed that the LTNP-04-1 *vpr* sequence was still predominant at those times (Fig. 1B). This increase in HIV-1 diversity might be related to an increase in viral fitness, which in turn is a determining factor in infection progression [29]–[32].

**Table 1:** Relative frequency of primary Vpr alleles^a^.

Samples	Vpr alleles	Number of clones/total number of sequenced clones (%)
Sample 1986	LTNP-86	40/40 (100)
Sample 1996	LTNP-96	40/40 (100)
Sample 2004	LTNP-04-1	23/40 (57.5)
	LTNP-04-2	6/40 (15)
	LTNP-04-3	3/40 (7.5)
	LTNP-04-4	3/40 (7.5)
	LTNP-04-5	5/40 (12.5)
Sample PR	PR1	18/42 (43)
	PR2	18/42 (43)
	PR3	6/42 (14)

a *vpr* genes were amplified by PCR from each PBMC sample (indicated by blue arrows in Fig. 1A), cloned into a shuttle plasmid, and DNA sequences from at least 40 independent clones were determined from each sample.

b samples 1986, 1996 and 2004 are from the LTNP patient (5071), and sample PR is from the progressor patient (1200).

c Vpr alleles correspond to the primary amino acid sequences reported in Fig. 1B.

Interestingly, *vpr* alleles from LTNP-86 and LTNP-04 samples all contained a R77Q substitution, previously reported as deleterious for Vpr proapoptotic activity [22], [23], whereas LTNP-96 showed an Arg at this position (Fig. 1B). In addition, Vpr LTNP-04-2 contained the Q65R substitution within the leucine-rich domain of Vpr, recently shown as deleterious for Vpr binding to the DCAF1 subunit of the Cul4a/DDB1 E3 ligase [12]–[17]. However, this *vpr* allele was found only in a minor virus population of the LTNP-04 PBMC sample (Table 1), and no correlation could be established between this mutation and viral load or CD4+ T-cell count. Regarding the *vpr* sequences isolated from the progressor patient, PR-2 had a substitution of Ser79 in Gly which is predicted to affect Vpr phosphorylation and its G2-arrest activity [33], [34]. Moreover, PR-3 showed a L64P substitution also described as precluding G2-arrest [35].

The LTNP-86, LTNP-96 and two predominant alleles from the 2004 sample (LTNP-04-1 and LTNP-04-2), as well as the three alleles of the progressor patient (PR-1, PR-2 and PR-3), were selected for further functional characterization.

### Subcellular localization of the primary Vpr proteins

**Figure 2:**
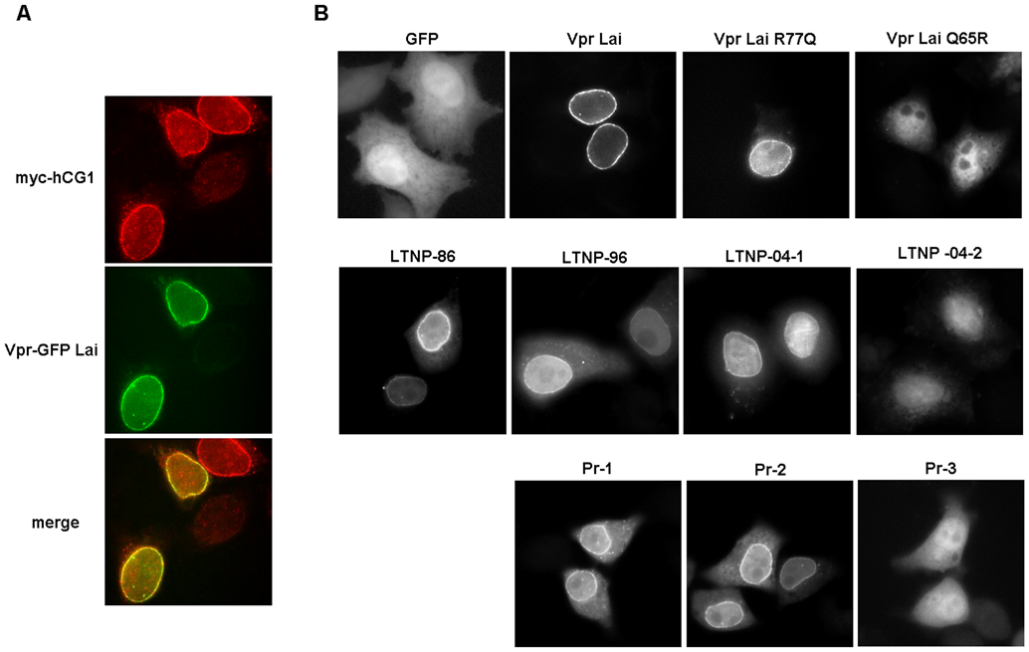
Cellular localization of Vpr proteins from the LTNP and PR patients. (A) Co-localization of VprLai and hCG1 at the NE. HeLa cells were transiently transfected with vectors for expression of VprLai fused to GPF together with hCG1 fused to the Myc tag. 16 h after transfection, cells were fixed and subcellular distribution of the fusion proteins was analyzed by epifluorescence microscopy. (B) Subcellular distribution of the indicated primary Vpr proteins from the LTNP and PR patients fused to GFP. Cells expressing GFP were used as a control.

HIV-1 Vpr primarily localizes in the nucleus, but also accumulates at the NE where it co-localizes with components of the nuclear pore complex, such as the nucleoporin hCG1 (Fig. 2A) (see Ref. [1] for review). A similar cellular distribution was revealed for the LTNP-86, -96 and -04-1 proteins, when expressed as Vpr-GFP fusions (Fig. 2B). Interestingly, the Vpr LTNP-04-2 protein, containing the Q65R substitution, as well as the Vpr*Lai*-GFP Q65R mutant, failed to accumulate at the NE (Fig. 2B), indicating that this residue participates in the proper localization of Vpr. When expressed as HA-tagged proteins, these Vpr variants similarly co-distributed in the cytoplasm and the nucleus, whereas wt HA-Vpr*Lai* was concentrated into the nucleus and at the NE (data not shown). This observation also indicates that the Vpr/DCAF1 interaction might be functionally related to the docking of Vpr at the NE.

Among Vpr proteins isolated from the progressor patient, the minor PR-3 allele (see on Table 1) was found equally distributed between the cytoplasm and the nucleus (Fig. 2B). However, we failed to detect the PR-3 protein in transfected T-cells (see below), suggesting that this variant was rather unstable.

### Interaction of primary variants with cellular partners of Vpr

**Figure 3:**
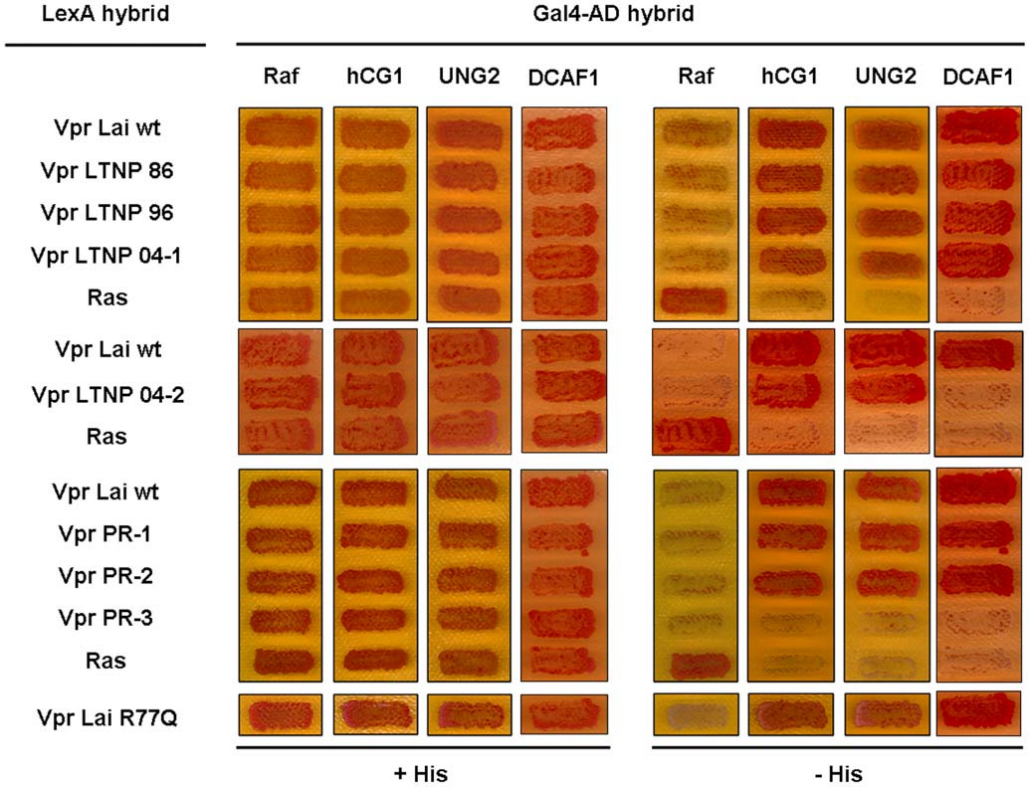
Interaction of the Vpr proteins from the LTNP and PR patients with hCG1, UNG2 and DCAF1 in the yeast two-hybrid system. The L40 reporter yeast strain expressing Vpr*Lai* or the indicated primary Vpr proteins from the LTNP and PR patients fused to LexA (LexA hybrid), in combination with either hCG1, UNG2 or DCAF1 fused to the Gal4 activation domain (Gal4AD hybrid), was analyzed for histidine auxotrophy. Growth in the absence of histidine indicates interaction between hybrid proteins.

Vpr properties have been related to its ability to interact with host cell partners, including the nucleoporin hCG1, the UNG2 enzyme and DCAF1. These cellular proteins were thus analyzed in the yeast two-hybrid system for interaction with primary Vpr variants isolated from both LTNP and PR patients. Most of the variants did interact with hCG1, UNG2 and DCAF1, as visualized by growth of yeast-transformed cells on medium without histidine (Fig. 3), arguing for a good conservation of the structural determinants required for Vpr functions. While no variant had substitution of the Trp54 residue required for UNG2 binding [4], [5], [35], LTNP-04-2 naturally contained the Q65R substitution and failed to interact with DCAF1, confirming the crucial role of this residue in mediating Vpr/DCAF1 interaction in the context of a primary Vpr variant [13], [15]. It is noteworthy that the residue 77 had no impact on Vpr binding to hCG1, UNG2 or DCAF1. Like Vpr*Lai* and Vpr*Lai*-R77Q, primary Vpr proteins with an Arg or a Gln similarly bound to these cellular proteins (Fig. 3). Finally, the negative results obtained with the PR-3 variant suggested that it was also poorly expressed in yeast cells.

### G2-arrest and cell death induction of Vpr variants

**Figure 4:**
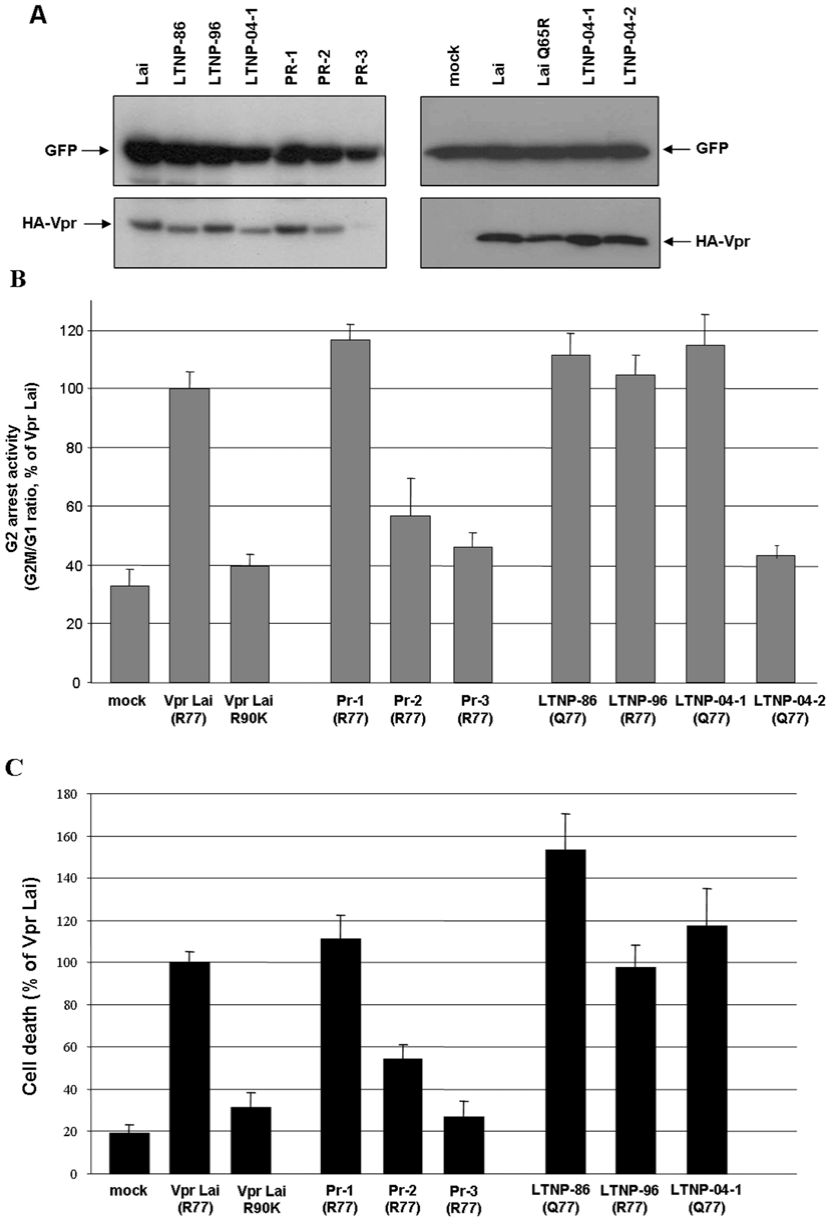
G2-arrest and pro-apoptotic activities of Vpr proteins from the LTNP and PR patients. HPB-ALL T lymphoid cells were co-transfected with vectors for expression of HA tagged Vpr*Lai* or the indicated primary Vpr proteins from the LTNP and PR patients, in combination with the GFP expression vector. A) Cellular expression of HA-tagged Vpr proteins. Lysates from HPB-ALL transfected cells were analyzed by western-blotting using anti-GFP and anti-HA antibodies. B) G2-arrest activity of primary Vpr proteins. 48 h after transfection, cells were fixed and the DNA content of GFP-positive cells was analyzed by flow cytometry after DNA staining with propidium iodide. Results are expressed as the G2M/G1 ratios relative to that of the VprLai. C) Pro-apoptotic activity of Vpr proteins. Cells co-expressing the GFP and HA-tagged Vpr proteins were assayed by flow cytometry 72 h following transfection for cell surface phosphatidylserine exposure using AnnexinV coupled to phycoerythrin. Values are means of three independent experiments; error bars represent one standard deviation from the mean.

Since the G2-arrest activity and apoptosis induction are the most documented effects of Vpr *in vitro* (reviewed in Refs [1], [36]), Vpr variants were assayed in T lymphocytes for these activities (Fig. 4). Consistent with our previous observations [37], Vpr-induced apoptosis of all variants (Fig. 4C) strictly paralleled the results obtained in the G2-arrest experiments (Fig. 4B). As expected, LTNP-04-2 Vpr, containing the Q65R substitution, failed to induce G2-arrest when compared to Vpr*Lai*. Similarly, PR-2 and PR-3 variants were less efficient than Vpr*Lai*. While the lack of activity of PR-3 was due to a problem of expression (Fig. 4A) that might be consequent to the presence of the structurally destabilizing L64P mutation [38], the reduced activity of PR-2 may be related to the S79G substitution, previously shown as deleterious for this activity [33], [34].

The results reported in Fig. 4 confirm that induction of G2-arrest and apoptosis by Vpr are functionally linked, even if others have suggested that the G2-arrest was not a prerequisite for induction of apoptosis [39]–[41]. Although it was proposed that the HIV-1 LTR was more active in the G2 phase [18], the significance of this cell cycle arrest during infection is still not well understood. Nevertheless, some studies have demonstrated that p24-positive cells isolated from infected individuals have a DNA content that is consistent with G2-arrest [40], indicating that further studies are needed to understand the role of this well-conserved Vpr function in vivo.

**Figure 5:**
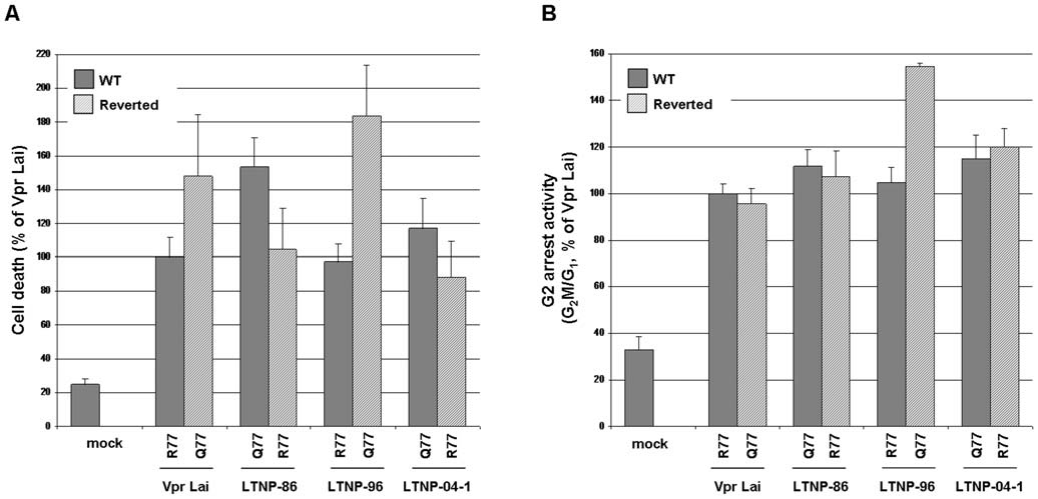
Impact of the residue 77 on pro-apoptotic and G2-arrest activities of Vpr LTNP variants. The Arg77 residue from VprLai and Vpr LTNP-96 was replaced by a Gln, whereas the Gln residue from LTNP-86 and LTNP-04-1 was replaced by an Arg. The wild-type (grey bars) and mutated (hatched bars) Vpr proteins were then analyzed for apoptosis (A) and G2-arrest (B) activities as described in Figure 4.

Interestingly, Vpr variants containing the R77Q substitution, namely LTNP-86 and LTNP-04-1, were even slightly more efficient for G-2 arrest and pro-apoptotic activities than those containing an Arg77, including LTNP-96 and PR-1 as well as the prototypic Vpr*Lai* (Fig. 4), arguing against a deleterious impact of the R77Q substitution on Vpr functions [22]. To further analyze the influence of the R77Q substitution on the proapoptotic activity of Vpr in the context of the LTNP alleles, we performed a detailed reverse mutational analysis. While the Gln77 found in LTNP-86 and LNTP-04-1 was mutated to Arg, the Arg77 found in LTNP-96 was mutated to Gln. As shown in Fig. 5A, the Q77R substitution of LTNP-86 and LNTP-04-1 resulted in a slightly reduced ability to induce apoptosis, whereas the R77Q LTNP-96 mutant was more active than the parental variant. Furthermore, a similar phenotype was observed when the R77Q substitution was introduced in Vpr*Lai*. Altogether, these data do not support previous observations on the critical role of Arg77 for the ability of Vpr to induce apoptosis [22]. Similarly, the residue in position 77 had no influence on Vpr-mediated G2-arrest (Fig. 5B). In contradiction with some reports [22], [23], we show that the R77Q substitution, found in the sequence of some *vpr* alleles from LTNPs, does not abolish the pro-apoptotic activity of Vpr. Instead, our data suggest that this substitution have a null to moderate positive impact on this activity, in agreement with recent reports showing that introduction of the R77Q mutation in HIV-1*NL4-3* Vpr results in a functional protein [37], [42].

One original finding of the present study relies on the identification, in the context of the natural infection, of a Vpr variant harboring the Q65R substitution previously reported as deleterious for DCAF1 binding and consequently for Vpr-induced cell cycle arrest [12]–[17]. Moreover, we now report that this mutation, in both a primary Vpr variant and the Vpr*Lai* Q65R mutant, abrogates Vpr docking at the NE, raising the possibility of a functional link between Vpr accumulation at the NE and its cytostatic activity. Indeed, it was reported that Vpr expression induced transient ruptures of the NE, resulting in a mixing of cytoplasmic and nuclear components that regulate the cell cycle [39]. As previously discussed [43], Vpr docking at the NE could constitute a prerequisite for binding to DCAF1 and subsequent induction of cell cycle arrest. This hypothesis is strengthened by the observation that almost all Vpr mutants described so far as disrupting the NE accumulation are altered as well for the G2-arrest activity [10], [19], [20], [44]. Alternatively, Vpr binding to DCAF1 might be required for proper docking of Vpr at the NE, where it could then exert its cytostatic activity by means of local interactions.

Although no correlation between Vpr functionality of dominant primary alleles and evolution of viral load during HIV-1 infection could be made from two unique patients, this could be achieved through large scale longitudinal studies investigating molecular and functional aspects of HIV-1 Vpr. In the present work, we used this rational approach to revisit, in the context of the natural infection, some aspects of Vpr molecular and functional properties that had been described so far by means of artificially created mutants. Our findings indicate that Vpr variants from either LTNP or progressor patients retain most of the functional features of the HIV-1 Vpr protein previously characterized in different *in vitro* systems. Most of the primary Vpr proteins displayed efficient cytostatic and pro-apoptotic activities when expressed in T lymphocytes. Although Vpr certainly constitutes an important determinant of virulence *in vivo*, the real contribution of both cytostatic and cytotoxic properties of the protein to the viral replication and pathogenesis during the course of HIV-1 infection remains unclear. In addition, our results also open new perspectives regarding a possible functional link between Vpr accumulation at the NE and its ability to interact with the DCAF1 subunit of the Cul4a/DDB1 E3 ligase.

## Materials and Methods

### HIV-1 infected Patients

Based on clinical, virological and immunological status (Fig. 1A), two patients with a well-documented natural history of infection (one non-progressor and one progressor) were selected from the HIV-1-infected patient cohort of the St-Antoine Hospital, Paris. These caucasian patients were infected in 1985 with subtype B viruses. The LTNP patient was still asymptomatic 22 years after contamination, in the absence of antiretroviral therapy, with relatively stable CD4 cell counts (>350 cells/mm3) and low levels of viral RNA for the first 10 years of infection. From 2000 to 2006, we observed an increase in the copy number of viral RNA followed by a decrease in the CD4 cell-count [45], [46]. Three samples of frozen peripheral blood mononuclear cells (PBMC) at different time points (1986, 1996, 2004, 2005 and 2006) were analyzed. Conversely, the progressor patient was infected in 1985 and rapidly treated by ZDV on the basis of a low level of CD4+ T lymphocytes (<200 cells/mm3); the PBMC sample analyzed herein was isolated in 1988, 2 years before the patient died of AIDS. All the blood samples were collected in a previous longitudinal study, approved by the ethics committee from the St-Antoine Hospital (Paris, France), with written informed consent from infected patients [45], [46].

### Viral DNA isolation and sequencing

PBMCs were isolated by Ficoll-hypaque density gradient centrifugation, and viral DNA was extracted using QIAamp blood extraction kit (QIAGEN). For each sample, 500 ng DNA were used to amplify *vpr* genes by two sequential PCR as previously described [22]. The final PCR product was sub-cloned into the pCR3.1 plasmid using the TA Cloning kit (Invitrogen) and subsequently transformed into DH5α Max Efficiency bacteria (Invitrogen). At least 40 clones were sequenced.

### Expression vectors

The primary *vpr* alleles were subcloned into expression vectors for further characterization. *Vpr* genes were thus amplified from the pCR3.1 constructs, using the following primers: VprBam-F-AGTCGGATCCATGGAACAAGCCCCAGAAGAC and VprXho-R-ACTGCTCGAGTCAGGATCTACTGGCTCCATT for subcloning into the pLex10 and pAS1B plasmids as previously described [43] , for expression of Vpr as a fusion to LexA or to the HA-tag in yeast and mammalian cells, respectively; VprXho-FACTGCTCGAGCTATGGAA CAAGCCCCAGAAGAC and VprBam-R(ΔTGA)ACTGGGATCCGGATCTACTGGCTCCATT for subcloning into pEGFP-N3 as described [43] for expression of Vpr as fusions to the N-terminal of the GFP. The vectors for yeast and mammalian expression of hCG1 and UNG2, and the vector for expression of VprR90K were previously described [9], [47]. The vector for yeast expression of DCAF1 was provided by F. Margottin-Goguet (Cochin Institute, Paris) [15]. Site-directed mutagenesis on residue 77 was performed by PCR using specific primers containing the desired mutations.

### Yeast two-hybrid

The L40 yeast reporter strain containing the *HIS3* LexA-inducible genewas cotransformed with the indicated LexABD and Gal4AD hybrid expression vectors and plated on selective medium as reported [4], [47]. Double transformants were patched on the same medium and replica plated on selective medium lacking histidine for auxotrophy analysis as described.

### Cell culture and transfections

HeLa cells and CD4-positive HPB-ALL T-cells were maintained as previously described [9], [48]. HeLa cells were transfected with the Vpr-GFP constructs alone or in combination with Myc-hCG1 expression vector using the calcium phosphate method as described [9]. HPB-ALL cells were electroporated as described [48] with the GFP expression vector as a transfection marker, and the HA-Vpr expression vectors.

### Immunofluorescence

18 h after transfection, HeLa cells were fixed with 4% paraformaldehyde (PFA) and permeabilized as described. [9]. Cells expressing Myc-hCG1 were permeabilized with 55 µg/ml digitonin (Sigma) and then fixed with 4% PFA [9]. Anti-Myc (9E10, Roche) and TexasRed-conjugated anti-mouse IgG (Jackson) were use as primary and secondary antibodies, respectively. Images were acquired with a Leica DMRB epifluorescence microscope equipped with a CCD camera (Princeton) controlled by Metamorph V5.0r6 software **(Universal Imaging Corp.)**.

### Cell cycle and apoptosis

48 h after transfection, half of HPB-ALL cells were collected and fixed with PFA 1% as described [43], [48]. Cells were then permeabilized in cold 70% ethanol and incubated with 200 µg/ml RNAse A and 50 µg/ml propidium iodide. 72 h after transfection, the remaining HPB-ALL cells were analyzed by flow cytometry for cell surface phosphatidylserines (PS) using phycoerythrin-conjugated annexin V (AnnexinV-PE, Bender MedSystems) as described [43], [48]. Cell cycle and PS-exposure profiles were analyzed on GFP-positive cells using a Cytomics FC 500 instrument (Beckman Coulter).

### Protein expression

HPB-ALL cells expressing HA-Vpr proteins together with GFP were lysed, separated by SDS-PAGE and transferred to a polyvinylidene difluoride (PVDF) Hybond-P membrane (GE Healthcare) as described [48]. Membranes were probed with mouse anti-GFP 7.1–13.1 (1814460, Roche) and rat anti-HA 3F10 (Roche) primary antibodies and with secondary HRP-coupled anti-mouse and anti-rat antibodies (Sigma).
